# Pilot study of an integrative telehealth group intervention for chronic pain

**DOI:** 10.1097/MD.0000000000041952

**Published:** 2025-03-21

**Authors:** Laura Jean Priorello, Jeannie Anne Sperry, Claire Ida Yee, Shweta Kapoor, David Clarence Patchett, Cynthia Oswald Townsend

**Affiliations:** a Department of Psychiatry and Psychology, Mayo Clinic Arizona, Scottsdale, AZ 85259, USA.

**Keywords:** chronic pain, cognitive behavioral therapy, mindfulness, psychotherapy, telehealth

## Abstract

The effectiveness of in-person psychological interventions for chronic pain populations has been widely studied. The current retrospective pilot study evaluates the effectiveness of a 10-week integrative telehealth pain group intervention consisting of cognitive, behavioral, mindfulness, and lifestyle strategies on anxiety, depression, pain catastrophizing, pain interference, and pain intensity. Participants at a large multidisciplinary hospital are referred internally from various medical departments within the hospital. The present study consists of data from 9 group cohorts from October 2020 to June 2022. The study included 86 patients, with 52 completing all 10 weeks of the intervention with post-questionnaire data available. Measures assessing anxiety, depression, pain catastrophizing, pain interference, and pain intensity at baseline and at the completion of the intervention. A series of paired samples t-tests were used to assess change in each outcome measure from baseline to after completion of the program. All the outcome variables including anxiety, depression, pain interference, pain intensity, and pain catastrophizing showed statistically significant reductions after the intervention compared to baseline assessments. There were no significant differences in any of the demographic variables (age, gender, ethnicity, education level) or chronic pain condition between participants who did and did not complete the program. Preliminary data suggest that this 10-week integrative telehealth pain group intervention significantly lowered anxiety, depression, pain catastrophizing, pain interference, and pain intensity from pre- to post-intervention. Larger, randomized controlled studies are needed to validate these results.

## 1. Introduction

The International Association for the Study of Pain (IASP) defines chronic pain as “an unpleasant sensory *and emotional experience* associated with, or resembling that associated with, actual or potential tissue damage, recently expanding their definition to include “pain is always a personal experience that is influenced to varying degrees by biological, psychological, and social factors.”^[1]^ According to a 2023 Center For Disease Control report, approximately 24.3% of adults in the United States (U.S.) experience chronic pain, with 8.5% acknowledging high-impact chronic pain (limiting life or work activities on most or every day) for at least 3 months.^[2]^ Likely due to its adverse impact on quality of life, daily activities, functioning, relationships, employment, and mood, chronic pain is 1 of the most widely studied medical conditions in the psychological literature and typically requires a multifactorial approach to management. The present study utilized a Cognitive behavioral therapy (CBT) approach combined with principles of mindfulness, movement, and lifestyle interventions (described in more detail below) in a telehealth delivery format to demonstrate the effectiveness and feasibility of this protocol for patients with chronic pain.

Cognitive behavioral therapy is a psychological intervention that can assist individuals with chronic pain in learning to manage their pain, restore functioning, and reduce the control that pain exerts over their lives.^[3]^ CBT is based on the cognitive model, which postulates that an individual’s perceptions/thoughts of a situation directly impact his or her emotional, behavioral, and physiological response(s); thus, patients learn how by increasing awareness of their pain-related distressing thoughts they can reduce the severity of painful symptoms and emotional reactions. Typical components of CBT include psychoeducation, cognitive restructuring, behavioral activation, relaxation training, problem solving, homework, treatment planning/goal setting, and relapse prevention.^[3]^

Cognitive behavioral therapy for chronic pain (CBT-CP) has demonstrated small to moderate effects on mood and pain-related outcomes. In a 2015 systematic review and meta-analysis examining 23 studies and 3300 patients with subacute and chronic low back pain, CBT-CP was significantly associated with improvements in both short- and long-term pain, disability, and quality of life, and was superior to waitlist control, usual care, and guideline-based active treatment.^[4]^ However, a recent systematic review of 75 randomized controlled trials showed that CBT-CP vs treatment as usual showed a small effect size for pain, disability, and distress and CBT vs active treatment showed few effects.^[5]^ Thus, given this evidence on CBT-CP, it is important that further investigations be conducted to identify ways to improve these effect sizes, such as supplementing CBT-CP with mindfulness-based or other evidence-based interventions. Additionally, several studies highlight the importance of CBT-CP in influencing neural changes and including a neuroscience education component in a chronic pain treatment program, as our group intervention does.^[6–8]^

Another effective intervention for chronic pain management identified in the literature is mindfulness-based stress reduction (MBSR), which is a mind-body, meditation-based therapy approach.^[9,10]^ Meta-analyses of MBSR covering a range of physical and mental disorders (chronic pain, cancer, heart disease, anxiety, depression, etc) have found medium to large effect sizes, and conclude that mindfulness-based interventions “might enhance general features of coping with distress and disability in everyday life, as well as under more extraordinary conditions of serious disorder or stress.”^[9,11]^ In a recent randomized controlled trial on a mindfulness-oriented recovery enhancement intervention vs supportive group therapy for co-occurring opioid misuse and chronic pain in primary care, researchers found that MORE led to greater improvements in pain severity and functional interference, and decreases in emotional distress, depressive symptoms, and real-time reports of opioid craving in daily life than supportive therapy; in addition, the MORE intervention led to large and statistically significant decreases in opioid misuse compared to supportive therapy.^[12]^

There is also increasing evidence on using both CBT and mindfulness training in patients with chronic pain. One study examined CBT and MBSR group interventions separately and found that the addition of MBSR offers significant benefit for chronic pain management.^[10]^ A 2016 randomized controlled trial on group therapy with adults with chronic low back pain found that treatment with CBT or MBSR resulted in greater improvement in pain and functional limitations at 26 weeks compared with usual care and there were no significant differences in outcomes between CBT and MBSR.^[13]^ To our knowledge, there are no published studies on MBSR teletherapy interventions for chronic pain management. To add to the above research, it makes sense to include a mindfulness component into our CBT-based pain management group.

Lastly, there is increasing research on telehealth-based psychological interventions for chronic pain management, as this platform can help aid in patient safety and reduce financial, time, and transportation barriers, as well as expanding treatment to a larger geographic area. In addition to a structured 1-on-one intervention, CBT for pain can be effectively provided in many other formats, including groups, web-based self-help programs, and via telephone.^[14–16]^ Results of a recent randomized controlled trial on the clinical and cost-effectiveness of videoconference-based CBT for chronic pain suggested that this intervention may reduce pain interference and disability, but not pain intensity; this method also demonstrated cost-effectiveness within a small sample size, though further replication using a larger sample size is needed for verification purposes.^[17]^ Another recent pilot study on a live teletherapy CBT pain management group found significant pre-post differences in pain-related interference and disability, but no changes in negative affect.^[18]^ This particular study only examined patients with chronic back pain using CBT principles with minimal emphasis on mindfulness components and/or mindful movement strategies. In addition, these researchers did not measure pain catastrophizing, which is an important outcome to assess given its impact on the cognition and emotional processing of patients with chronic pain. Our current study will address these gaps by including a more comprehensive sample of chronic pain patients, an entire group session devoted to mindfulness components in addition to a 30-minute mindful movement practice led by a certified yoga instructor, and pain catastrophizing as an outcome variable. In addition, the present study adds to this research on the benefit and feasibility of telehealth psychology interventions for chronic pain management.

In light of the above-mentioned research, we conducted a pilot study on a 10-week/session (90-minute) chronic pain management group offered virtually via a Zoom videoconference platform across 9 cohorts from October 14, 2020, to June 7, 2022. Patients were assessed for anxiety, depression, pain catastrophizing, pain interference, and pain intensity at baseline, and at the completion of the 10-week intervention. We hypothesize that our results will demonstrate significant improvements in all primary outcome variables (anxiety, depression, pain interference, intensity, and catastrophizing).

## 2. Methods

### 2.1. Study design and intervention

This pilot is a retrospective study that was performed at Mayo Clinic in Arizona and was approved by the Institutional Review Board of the hospital system (#22-008387). This study examined a virtual integrated CBT and mindfulness group intervention comparing pretreatment with immediate posttreatment self-reported measures to assess changes associated with the intervention. The virtual integrated group intervention consisted of 10 sessions over a consecutive 10-week period, each 90-minutes long; each group was closed to maintain confidentiality/privacy. The sample consists of 86 patients, with 52 completing all 10 weeks of the intervention with available post-questionnaire data.

The group sessions consisted of a combination of psychoeducation with Power Point presentations, discussion, and in vivo practice of therapy techniques (e.g., relaxation training, cognitive restructuring, goal setting, etc). Approximately 1 to 2 weeks prior to the start of the group, there is an orientation session to discuss the nature, expectations, and purpose of the group. The group topics over the 10 sessions include: education on the biology of pain, relaxation and stress relief exercises, cognitive restructuring, attention diversion techniques and distress tolerance skills, mindfulness and imagery skills, sleep management, activity pacing and goal setting, managing pain flares and relapse prevention, lifestyle interventions, and mindful movement/meditation. During the “Lifestyle Interventions for Pain Management” session, participants watched a video recorded specifically for this group by a physician specializing in family and integrative medicine focused on diet, nutrition, physical activity, alternative pain interventions, etc. The “Mindful Movement/Meditation” group session was instructed live by a Certified Lu Jong Tibetan Healing Yoga Teacher at Mayo Clinic Arizona. All other sessions were facilitated by a licensed and board-certified clinical health psychologist. As noted above, there is also a group session on mindfulness practices for pain management that included psychoeducation on mindfulness and an instructor-led basic mindfulness meditation practice. These 2 mindfulness-oriented sessions and the lifestyle intervention session described above create the integrated components of the CBT intervention.

### 2.2. Participants

Participants are referred internally from various medical departments within Mayo Clinic Arizona (i.e., Pain Medicine, Neurology, Family Medicine, Integrative Medicine, Psychiatry/Psychology, Community Internal Medicine, Gynecology, etc) and referring providers receive a group flyer with instructions on how to refer patients to the group. Participants have a wide range of chronic pain conditions (see more detail in demographic information below).

Inclusion criteria consists of patients with a primary pain diagnosis, willingness to learn how to effectively manage his/her chronic pain using evidence-based psychological interventions in a supportive group atmosphere, English fluency, adults aged 18 and over, and ability to attend/commit to at least 8 90-minute treatment sessions. Exclusion criteria consists of patients who are primarily seeking medication or medical intervention, patients who have current severe mental health symptoms (suicidal ideation, psychosis, severe depression/anxiety), patients with significant disruptive and/or impulsive behaviors that might hinder the therapeutic group process and effectiveness, and patients with gross cognitive impairment.

### 2.3. Measures

Existing research shows that chronic pain is related to depression, anxiety, interference in daily activities, and pain catastrophizing.^[5,19]^ Thus, prior to the first and last day of the group, pain group participants complete 4 measurements to assess for pre- and post- group changes: the Patient Health Questionnaire-9 (PHQ-9),^[20,21]^ a measure of depression symptoms; the Generalized Anxiety Disorder-7 Questionnaire (GAD-7),^[22]^ a measure of anxiety symptoms; the PEG, a very brief 3-item scale derived from the Brief Pain Inventory that assesses average pain intensity (P), interference with enjoyment of life (E), and interference with general activity (G)^[23]^; and the pain catastrophizing scale,^[18]^ a measure of one’s tendency to magnify the threat value of a pain stimulus and feel helpless in the presence of pain.^[24]^ Each of these measurements have demonstrated good psychometrics in chronic pain populations.

### 2.4. Statistical methods

Demographic variables were described using mean and standard deviation (SD), while continuous variables were described using frequencies and percentages. Differences between those who completed the program compared to those who did not were examined using chi-square tests and independent samples t-tests. Distributions, and means and SDs were used to determine a normal distribution for all variables. A series of paired samples t-tests were used to assess change in each outcome measure from baseline to after the 10-week intervention. Pain intensity was assessed as individual items: average pain intensity, life interference, and activity interference, with a separate test for each measure. Sample size was determined by assuming a medium effect size (Cohen *d* = 0.5) and for a paired samples *t*-test. The sample for this pilot study surpassed the size of 44 patients needed for 90% power. *P*-values < .05 were considered statistically significant. R version 4.1.2 was used for statistical analyses. The Bonferroni correction method was used to control for the number of tests looking at differences in demographic variables, and a separate correction was used for the number of tests looking at change over time in all the outcome measures. After the adjustment, all results remained statistically significant. Of note, a statistician assisted with all statistical methods.

## 3. Results

### 3.1. Descriptives

Overall, most of the patients were female (82.6%) and White/not Hispanic or Latino (86%). 42% of the sample had an associate or high school equivalent diploma with 56.9% having at least a bachelor’s degree. The average age of the sample was 52.7 years (SD = 14.897). Of the 86 patients, 52 completed the entire 10-week intervention. There were no differences in any of the demographic variables based on completion status (see Table 1). Similarly, none of the outcome variables differed at baseline between program completers and non-completers.

**Table 1:** Patient characteristics by completion status.

	Incomplete (N = 34)	Complete (N = 52)	Total Sample (N = 86)	*P*-value
Age				
*M* (SD)	50.912 (15.212)	53.885 (14.716)	52.709 (14.897)	.369*
Range	22 to 80	21 to 88	21- 88	
Gender				
Female	26 (76.5%)	45 (86.5%)	71 (82.6%)	.229†
Male	8 (23.5%)	7 (13.5%)	15 (17.4%)	
Ethnicity				
White/Not Hispanic or Latino	29 (85.3%)	45 (86.5%)	74 (86.0%)	.454†
Hispanic or Latino	4 (11.8%)	7 (13.5%)	11 (12.8%)	
Did not disclose	1 (2.9%)	0 (0.0%)	1 (1.2%)	
Education level				
12th grade/GED	6 (17.6%)	5 (9.6%)	11 (12.8%)	.486†
Associates/some college	10 (29.4%)	15 (28.8%)	25 (29.1%)	
Bachelors	13 (38.2%)	17 (32.7%)	30 (34.9%)	
Masters	3 (8.8%)	12 (23.1%)	15 (17.4%)	
Professional degree/Doctorate	2 (5.9%)	2 (3.8%)	4 (4.7%)	
Missing	0 (0.0%)	1 (1.9%)	1 (1.2%)	
Anxiety (GAD-7)				
*M* (SD)	9.481 (6.272)	7.923 (5.346)	8.456 (5.688)	.251*
Range	0.000 to 21.000	0.000 to 21.000	0.000 to 21.000	
Missing	7	0	7	
Depression (PHQ-9)				
*M* (SD)	10.458 (6.338)	8.808 (5.841)	9.329 (6.010)	.269*
Range	2 to 24	0 to 22	0 to 24	
Missing	10	0	10	
Pain Catastrophizing (PCS)				
*M* (SD)	25.731 (14.010)	23.904 (11.122)	24.513 (12.100)	.533*
Range	2 to 52	5 to 48	2 to 52	
Missing	8	0	8	
Average pain				
*M* (SD)	6.864 (2.007)	6.538 (1.820)	6.635 (1.870)	.498*
Range	3 to 10	2 to 10	2 to 10	
Missing	12	0	12	
Life interference				
*M* (SD)	7.154 (2.461)	6.558 (2.697)	6.756 (2.620)	.347*
Range	1 to 10	0 to 10	0 to 10	
Missing	8	0	8	
Activity interference				
*M* (SD)	6.923 (2.544)	6.431 (2.722)	6.597 (2.657)	.446*
Range	2 to 10	0 to 10	0 to 10	
Missing	8	1	9	

GAD-7 = generalized anxiety disorder-7 questionnaire, PHQ-9 = patient health questionnaire-9.

* Independent samples *t*-test.

† Pearson Chi-squared test.

### 3.2. Chronic pain diagnoses

The most common pain diagnoses across the sample were chronic migraine (N = 42, 48.8%), and chronic pain syndrome (N = 31, 36%). On average patients had 2 pain diagnoses (SD = 1.153) (see Table S1, Supplemental Digital Content, http://links.lww.com/MD/O579). There were no differences between the 2 groups (completers vs non-completers) in whether there was a higher proportion of any diagnosis (see Table S2, Supplemental Digital Content, http://links.lww.com/MD/O580). Similarly, the 2 groups did not differ in the number of pain diagnoses they were diagnosed with.

### 3.3. Outcome measures

Means and SDs for all outcome measures are presented both at baseline and at follow-up after the 10-week intervention for all participants who completed the intervention (see Table 2).

**Table 2:** Outcome measures for those who completed the intervention.

	Baseline	Follow-up
Anxiety		
*M* (SD)	7.923 (5.346)	5.519 (5.020)
Range	0.000 to 21.000	0.000 to 21.000
Depression		
*M* (SD)	8.808 (5.841)	6.212 (4.816)
Range	0.000 to 22.000	0.000 to 17.000
Pain catastrophizing		
*M* (SD)	23.904 (11.122)	17.904 (10.408)
Range	5.000 to 48.000	0.000 to 46.000
Average pain		
*M* (SD)	6.538 (1.820)	5.423 (2.199)
Range	2.000 to 10.000	0.000 to 10.000
Life interference		
*M* (SD)	6.558 (2.697)	4.250 (2.685)
Range	0.000 to 10.000	0.000 to 10.000
Activity interference		
*M* (SD)	6.431 (2.722)	4.673 (2.720)
Range	0.000 to 10.000	0.000 to 10.000

Note: N = 52.

### 3.4. Analysis

Paired samples *t*-tests were used to determine whether there were differences between baseline and after the intervention for participants who completed an assessment both at baseline and after the 10-week intervention. On average, participants reported significantly less anxiety (*t* = −4.719, df = 54, *P* = <.0001) and depression (*t* = −4.96, df = 51, *P* = <.0001) after the intervention (Table 3). Similarly, pain catastrophizing (*t* = −5.047, df = 50, *P* = <.0001) and each type of pain intensity (Average pain: *t* = −4.495, df = 50, *P* = <.0001; Life interference: *t* = −6.492, df = 50, *P* = <.0001; Activity interference: *t* = −5.285, df = 50, *P* = <.0001) were significantly lower after the intervention compared to the baseline assessment.

**Table 3:** Results of paired samples *T*-tests for all outcome measures.

Variable	*M*_*B*_ - *M*_*P*_	*t*	*df*	*P*	95% CI [lower level, upper level]
Anxiety	−2.273	−4.719	54	<.0001	[−3.238 to −1.307]
Depression	−2.596	−4.96	51	<.0001	[−3.647 to −1.545]
Pain catastrophizing	−6.000	−5.047	50	<.0001	[−8.387 to −3.613]
Average pain	−1.115	−4.495	50	<.0001	[−1.614 to −0.617]
Life interference	−2.308	−6.492	50	<.0001	[−3.021 to −1.594]
Activity interference	−1.784	−5.285	50	<.0001	[−2.462 to –1.106]

Note: _**B**_ indicates mean at baseline; _P_ indicates mean post-intervention.

## 4. Discussion

To our knowledge, this is the first study to evaluate an integrative (CBT plus mindfulness plus lifestyle interventions) telehealth chronic pain group intervention assessing the variables of anxiety, depression, pain catastrophizing, pain interference, and pain intensity. Clinically, these findings highlight that participation in this integrative telehealth intervention for chronic pain was associated with significant improvements across all outcome variables.

This pilot study examined the preliminary efficacy of a 10-session (weekly) integrative telehealth intervention for chronic pain in a large hospital setting. The group content consisted of cognitive, behavioral, mindfulness, and lifestyle interventions for chronic pain management. Nine group cohorts were assessed on the outcome variables of anxiety, depression, pain catastrophizing, pain interference, and pain intensity from pre- to post-intervention. Our sample consisted of primarily female (82.6%), middle-aged (average 52.7 years (SD = 14.897)), White/not Hispanic or Latino (86%) individuals with at least a bachelor’s degree (56.9%), which appears to be representative of our greater hospital patient population. Our sample demographics is also similar to existing population-based research on gender differences in chronic pain consistently demonstrating greater pain prevalence with women reporting more than men.^[25]^ In addition, the majority (48.8%) of the participants had a primary diagnosis of chronic migraine, which is consistent with previous research on comprehensive pain rehabilitation center samples showing that the majority of patients referred to such programs have primary headache diagnoses.^[26]^ Within this sample of mostly college-educated females with chronic migraine diagnosis, results showed significant reductions on anxiety, depression, pain catastrophizing, pain interference, and pain intensity scores after the intervention compared to baseline assessments.

As mentioned above, existing research on videoconference-based CBT for chronic pain has suggested that this intervention reduces pain interference but not pain intensity or negative affect.^[17,18]^ However, the current study demonstrates that our integrative telehealth intervention does reduce pain intensity, in addition to depression and anxiety symptoms. Thus, results of our study highlight the importance of including mindfulness and lifestyle interventions into a CBT framework, and the benefit and feasibility of this videoconference-based intervention on reducing depression, anxiety, and pain intensity. In addition, in light of the limited, mixed research on combined CBT and mindfulness training in patients with chronic pain, this study illustrates the importance of including both of these strategies into an integrative chronic pain group intervention.

Several limitations exist in this study that are important to mention. First, this was not a randomized controlled trial and instead was a retrospective analysis, so patients did not sign an initial consent to commit to the completion of the group. Thus, we were unable to control for nonspecific and confounding factors, potentially limiting the generalizability of the results. Future and larger randomized, controlled trials are needed to validate these results. Second, although there was no difference in groups based on attrition status, this study had a large attrition rate, further affecting the validity of the results. Possible reasons for this attrition rate might be: patient hospitalizations or other complex illnesses preventing them from completing the group, patients were not identified as “completers” unless they completed the post-questionnaires; thus, some may have completed the 10-week intervention, but we do not have their data available, family or other psychosocial stressors arose, preventing completion of the 10-week intervention, this was not a randomized controlled trial and instead was a retrospective analysis; thus, patients did not sign an initial consent to commit to the completion of the group. Despite the attrition rate, this study demonstrates the acceptability of this virtual integrative intervention for chronic pain populations. More large-scale studies are needed examining various types of chronic pain conditions to examine the effectiveness of this program. Third, the lack of follow-up data limits our ability to assess the longevity of treatment effects; more long-term follow-up studies should be conducted in the future. Fourth, the pain intensity and pain interference data are based on single-item assessments, which may limit the validity of these findings. Fifth, due to the retrospective nature of this study, we are unable to gather data on any concurrent medical interventions or changes in medications that may have occurred over the course of the 10-week intervention. Further, we were unable to gather enough quality data for patient satisfaction to report in this study, which may have provided further support for the telehealth format of this group. Lastly, as discussed above, although not a significant finding, this sample is heavily weighted towards females with primary chronic migraine diagnosis and those with at least a bachelor’s degree education; males and lower educated individuals represented a decent number of non-completers. Thus, we cannot fully generalize the results to males or those with lower education or other chronic pain conditions, and future studies are needed to examine this intervention’s efficacy in these groups.

## 5. Conclusions

Despite the above-mentioned limitations, this virtual integrative chronic pain management group demonstrated the possibility of effectiveness and feasibility of such a delivery format and protocol for patients with chronic pain. The nature of this telehealth group provides increased access to evidence-based care, such as for individuals with compromised immune systems or disabilities, social anxiety, financial or transportation difficulties, and professional time away from work. During the coronavirus disease-19 pandemic in particular, this virtual group allowed individuals to receive consistent support and treatment needed without having to risk their health and safety. Further, as this virtual group intervention reduced mental health outcomes of anxiety and depression, it may have alleviated the prevalent anxiety and depression associated with the pandemic itself.

In summary, results of this preliminary study show that an integrative videoconference-based group intervention consisting of CBT, mindfulness, and lifestyle strategies can significantly reduce anxiety, depression, pain catastrophizing, pain interference, and pain intensity across chronic pain populations, with the majority of participants being at least college-educated females with chronic migraine. Thus, this program is a valuable addition to regular pain management treatment outcome studies and is recommended to be included as an adjunct to medical care. Further research is needed using randomized controlled trials, larger sample sizes, and follow-up assessments to extend and validate these results to chronic pain populations.

## Author contributions

**Conceptualization:** Laura Jean Priorello, Jeannie Anne Sperry, Shweta Kapoor, Cynthia Oswald Townsend.

**Data curation:** Laura Jean Priorello, Claire Ida Yee.

**Formal analysis:** Laura Jean Priorello, Claire Ida Yee.

**Investigation:** Laura Jean Priorello, Claire Ida Yee.

**Methodology:** Laura Jean Priorello, Shweta Kapoor, Claire Ida Yee, Cynthia Oswald Townsend.

**Project administration:** Laura Jean Priorello.

**Software:** Claire Ida Yee.

**Writing – original draft:** Laura Jean Priorello, Jeannie Anne Sperry, Shweta Kapoor, Claire Ida Yee, Cynthia Oswald Townsend.

**Writing – review & editing:** Laura Jean Priorello, Jeannie Anne Sperry, Shweta Kapoor, Claire Ida Yee, David Patchett, Cynthia Oswald Townsend.

## Supplementary Material

SUPPLEMENTARY MATERIAL

## References

[1] Raja SN Carr DB Cohen M. The revised International Association for the Study of Pain definition of pain: concepts, challenges, and compromises. Pain. 2020;161:1976–82. 32694387 10.1097/j.pain.0000000000001939 PMC7680716

[2] Lucas JW Sohi I. Chronic pain and high-impact chronic pain in U.S. adults, 2023. NCHS Data Brief. 2024:CS355235. 10.15620/cdc/169630 PMC11726267 39751180

[3] King R. Cognitive therapy of depression. Beck Aaron Rush John Shaw Brian Emery Gary. New York: Guilford, 1979. Aust N Z J Psychiatry. 2002;36:272–5. 10.1046/j.1440-1614.2002.t01-4-01015.x 11982560

[4] Richmond H Hall AM Copsey B. The effectiveness of cognitive behavioral treatment for non-specific low back pain: a systematic review and meta-analysis. PLoS One. 2015;10:e0134192. 26244668 10.1371/journal.pone.0134192 PMC4526658

[5] Williams ACC Fisher E Hearn L Eccleston C. Psychological therapies for the management of chronic pain (excluding headache) in adults. Cochrane Database Syst Rev. 2020;8:CD007407. 32794606 10.1002/14651858.CD007407.pub4 PMC7437545

[6] Jensen KB Kosek E Wicksell R. Cognitive behavioral therapy increases pain-evoked activation of the prefrontal cortex in patients with fibromyalgia. Pain. 2012;153:1495–503. 22617632 10.1016/j.pain.2012.04.010

[7] Seminowicz DA Shpaner M Keaser ML. Cognitive-behavioral therapy increases prefrontal cortex gray matter in patients with chronic pain. J Pain. 2013;14:1573–84. 24135432 10.1016/j.jpain.2013.07.020 PMC3874446

[8] Yoshino A Okamoto Y Okada G. Changes in resting-state brain networks after cognitive–behavioral therapy for chronic pain. Psychol Med. 2018;48:1148–56. 28893330 10.1017/S0033291717002598

[9] Grossman P Niemann L Schmidt S Walach H. Mindfulness-based stress reduction and health benefits. A meta-analysis. J Psychosom Res. 2004;57:35–43. 15256293 10.1016/S0022-3999(03)00573-7

[10] Khoo EL Small R Cheng W. Comparative evaluation of group-based mindfulness-based stress reduction and cognitive behavioral therapy for the treatment and management of chronic pain: a systematic review and network meta-analysis. Evid Based Ment Health. 2019;22:26–35. 30705039 10.1136/ebmental-2018-300062 PMC10270397

[11] Baer RA. Mindfulness training as a clinical intervention: a conceptual and empirical review. Clin Psychol. 2003;10:125–43.

[12] Garland EL Hanley AW Nakamura Y. Mindfulness-oriented recovery enhancement vs supportive group therapy for co-occurring opioid misuse and chronic pain in primary care: a randomized clinical trial. JAMA Intern Med. 2022;182:407–17. 35226053 10.1001/jamainternmed.2022.0033 PMC8886485

[13] Cherkin DC Sherman KJ Balderson BH. Effect of mindfulness-based stress reduction vs cognitive behavioral therapy or usual care on back pain and functional limitations in adults with chronic low back pain: a randomized clinical trial. JAMA. 2016;315:1240–9. 27002445 10.1001/jama.2016.2323 PMC4914381

[14] Dear BF Gandy M Karin E. The pain course: 12- and 24-month outcomes from a randomized controlled trial of an internet-delivered pain management program provided with different levels of clinician support. J Pain. 2018;19:1491–503. 30099209 10.1016/j.jpain.2018.07.005

[15] McBeth J Prescott G Scotland G. Cognitive behavior therapy, exercise, or both for treating chronic widespread pain. Arch Intern Med. 2012;172:48–57. 22082706 10.1001/archinternmed.2011.555

[16] Sander LB Paganini S Terhorst Y. Effectiveness of a guided web-based self-help intervention to prevent depression in patients with persistent back pain: the PROD-BP randomized clinical trial. JAMA Psychiatry. 2020;77:1001–11. 32459348 10.1001/jamapsychiatry.2020.1021 PMC7254449

[17] Taguchi K Numata N Takanashi R. Clinical effectiveness and cost-effectiveness of videoconference-based integrated cognitive behavioral therapy for chronic pain: randomized controlled trial. J Med Internet Res. 2021;23:e30690. 34813489 10.2196/30690 PMC8663446

[18] Mariano TY Wan L Edwards RR Lazaridou A Ross EL Jamison RN. Online group pain management for chronic pain: preliminary results of a novel treatment approach to teletherapy. J Telemed Telecare. 2021;27:209–16. 31431133 10.1177/1357633X19870369

[19] Sullivan MJL Bishop SR Pivik J. The pain catastrophizing scale: development and validation. Psychol Assess. 1995;7:524–32.

[20] Kroenke K Spitzer RL Williams JB. The PHQ-9: validity of a brief depression severity measure. J Gen Intern Med. 2001;16:606–13. 11556941 10.1046/j.1525-1497.2001.016009606.x PMC1495268

[21] Gilbody S Richards D Brealey S Hewitt C. Screening for depression in medical settings with the Patient Health Questionnaire (PHQ): a diagnostic meta-analysis. J Gen Intern Med. 2007;22:1596–602. 17874169 10.1007/s11606-007-0333-y PMC2219806

[22] Spitzer RL Kroenke K Williams JB Löwe B. A brief measure for assessing generalized anxiety disorder: the GAD-7. Arch Intern Med. 2006;166:1092–7. 16717171 10.1001/archinte.166.10.1092

[23] Krebs EE Lorenz KA Bair MJ. Development and initial validation of the PEG, a three-item scale assessing pain intensity and interference. J Gen Intern Med. 2009;24:733–8. 19418100 10.1007/s11606-009-0981-1 PMC2686775

[24] Osman A Barrios FX Gutierrez PM Kopper BA Merrifield T Grittmann L. The pain catastrophizing scale: further psychometric evaluation with adult samples. J Behav Med. 2000;23:351–65. 10984864 10.1023/a:1005548801037

[25] Bartley EJ Fillingim RB. Sex differences in pain: a brief review of clinical and experimental findings. Br J Anaesth. 2013;111:52–8. 23794645 10.1093/bja/aet127 PMC3690315

[26] Bruce BK Townsend CO Hooten WM Rome JD Moon JS Swanson JW. Chronic pain rehabilitation in chronic headache disorders. Curr Pain Headache Rep. 2009;13:67–72. 19126375 10.1007/s11916-009-0014-0

