# Supplementary material for: Pilot study of an integrative telehealth group intervention for chronic pain
Source: Medicine (Baltimore). 2025 Mar 21;104(12):e41952. doi: 10.1097/MD.0000000000041952 (PMC11936548; doi:10.1097/MD.0000000000041952)
Supplement: SUPPLEMENTARY MATERIAL [file medi-104-e41952-s001.docx]

Supplementary Table 1: Pain Diagnoses in Overall Sample

|  | Overall (N=86) |
| --- | --- |
| Fibromylagia |  |
| - Absent | 77 (89.5%) |
| - Present | 9 (10.5%) |
| Chronic migraine |  |
| - Absent | 44 (51.2%) |
| - Present | 42 (48.8%) |
| Neuropathic pain |  |
| - Absent | 77 (89.5%) |
| - Present | 9 (10.5%) |
| Chronic back |  |
| - Absent | 76 (88.4%) |
| - Present | 10 (11.6%) |
| Chronic low back |  |
| - Absent | 74 (86.0%) |
| - Present | 12 (14.0%) |
| Myofascial pain |  |
| - Absent | 80 (93.0%) |
| - Present | 6 (7.0%) |
| Cervical pain |  |
| - Absent | 81 (94.2%) |
| - Present | 5 (5.8%) |
| Central pain syndrome |  |
| - Absent | 84 (97.7%) |
| - Present | 2 (2.3%) |
| Chronic pain syndrome |  |
| - Absent | 55 (64.0%) |
| - Present | 31 (36.0%) |
| Generalized pain |  |
| - Absent | 84 (97.7%) |
| - Present | 2 (2.3%) |
| Chronic abdominal |  |
| - Absent | 78 (90.7%) |
| - Present | 8 (9.3%) |
| Neck pain |  |
| - Absent | 80 (93.0%) |
| - Present | 6 (7.0%) |
| Spondylosis cervical without myelopathy |  |
| - Absent | 85 (98.8%) |
| - Present | 1 (1.2%) |
| Phantom limb pain |  |
| - Absent | 85 (98.8%) |
| - Present | 1 (1.2%) |
| Pelvic pain |  |
| - Absent | 79 (91.9%) |
| - Present | 7 (8.1%) |
| Trigeminal Neuralgia |  |
| - Absent | 83 (96.5%) |
| - Present | 3 (3.5%) |
| Ankylosing spondylitis |  |
| - Absent | 85 (98.8%) |
| - Present | 1 (1.2%) |
| Chronic fatigue |  |
| - Absent | 81 (94.2%) |
| - Present | 5 (5.8%) |
| Polyarthralgia |  |
| - Absent | 84 (97.7%) |
| - Present | 2 (2.3%) |
| Leg pain |  |
| - Absent | 84 (97.7%) |
| - Present | 2 (2.3%) |
| Shoulder pain |  |
| - Absent | 83 (96.5%) |
| - Present | 3 (3.5%) |
| Hip pain |  |
| - Absent | 85 (98.8%) |
| - Present | 1 (1.2%) |
| Esophageal pain |  |
| - Absent | 85 (98.8%) |
| - Present | 1 (1.2%) |
| Chest pain |  |
| - Absent | 85 (98.8%) |
| - Present | 1 (1.2%) |
| Periumbilical |  |
| - Absent | 85 (98.8%) |
| - Present | 1 (1.2%) |
| Rheumatoid arthritis |  |
| - Absent | 85 (98.8%) |
| - Present | 1 (1.2%) |
| Complex regional pain syndrome |  |
| - Absent | 85 (98.8%) |
| - Present | 1 (1.2%) |
| Condition sum |  |
| - Mean (SD) | 2.012 (1.153) |
| - Range | 1.000 - 6.000 |

# 
