# Supplementary material for: Pilot study of an integrative telehealth group intervention for chronic pain
Source: Medicine (Baltimore). 2025 Mar 21;104(12):e41952. doi: 10.1097/MD.0000000000041952 (PMC11936548; doi:10.1097/MD.0000000000041952)
Supplement: SUPPLEMENTARY MATERIAL [file medi-104-e41952-s002.docx]

Supplementary Table 2: Pain Diagnoses by Complete vs Not Complete

|  | Not Complete (N=34) | Complete (N=52) | Total (N=86) | p value |
| --- | --- | --- | --- | --- |
| Fibromylagia |  |  |  | 0.688 ^1^ |
| - Absent | 31 (91.2%) | 46 (88.5%) | 77 (89.5%) |  |
| - Present | 3 (8.8%) | 6 (11.5%) | 9 (10.5%) |  |
| Chronic migraine |  |  |  | 0.862 ^1^ |
| - Absent | 17 (50.0%) | 27 (51.9%) | 44 (51.2%) |  |
| - Present | 17 (50.0%) | 25 (48.1%) | 42 (48.8%) |  |
| Neuropathic pain |  |  |  | 0.688 ^1^ |
| - Absent | 31 (91.2%) | 46 (88.5%) | 77 (89.5%) |  |
| - Present | 3 (8.8%) | 6 (11.5%) | 9 (10.5%) |  |
| Chronic back |  |  |  | 0.974 ^1^ |
| - Absent | 30 (88.2%) | 46 (88.5%) | 76 (88.4%) |  |
| - Present | 4 (11.8%) | 6 (11.5%) | 10 (11.6%) |  |
| Chronic low back |  |  |  | 0.424 ^1^ |
| - Absent | 28 (82.4%) | 46 (88.5%) | 74 (86.0%) |  |
| - Present | 6 (17.6%) | 6 (11.5%) | 12 (14.0%) |  |
| Myofascial pain |  |  |  | 0.747 ^1^ |
| - Absent | 32 (94.1%) | 48 (92.3%) | 80 (93.0%) |  |
| - Present | 2 (5.9%) | 4 (7.7%) | 6 (7.0%) |  |
| Cervical pain |  |  |  | 0.983 ^1^ |
| - Absent | 32 (94.1%) | 49 (94.2%) | 81 (94.2%) |  |
| - Present | 2 (5.9%) | 3 (5.8%) | 5 (5.8%) |  |
| Central pain syndrome |  |  |  | 0.247 ^1^ |
| - Absent | 34 (100.0%) | 50 (96.2%) | 84 (97.7%) |  |
| - Present | 0 (0.0%) | 2 (3.8%) | 2 (2.3%) |  |
| Chronic pain syndrome |  |  |  | 0.423 ^1^ |
| - Absent | 20 (58.8%) | 35 (67.3%) | 55 (64.0%) |  |
| - Present | 14 (41.2%) | 17 (32.7%) | 31 (36.0%) |  |
| Generalized pain |  |  |  | 0.759 ^1^ |
| - Absent | 33 (97.1%) | 51 (98.1%) | 84 (97.7%) |  |
| - Present | 1 (2.9%) | 1 (1.9%) | 2 (2.3%) |  |
| Chronic abdominal |  |  |  | 0.902 ^1^ |
| - Absent | 31 (91.2%) | 47 (90.4%) | 78 (90.7%) |  |
| - Present | 3 (8.8%) | 5 (9.6%) | 8 (9.3%) |  |
| Neck pain |  |  |  | 0.587 ^1^ |
| - Absent | 31 (91.2%) | 49 (94.2%) | 80 (93.0%) |  |
| - Present | 3 (8.8%) | 3 (5.8%) | 6 (7.0%) |  |
| Spondylosis cervical without myelopathy |  |  |  | 0.214 ^1^ |
| - Absent | 33 (97.1%) | 52 (100.0%) | 85 (98.8%) |  |
| - Present | 1 (2.9%) | 0 (0.0%) | 1 (1.2%) |  |
| Phantom limb pain |  |  |  | 0.416 ^1^ |
| - Absent | 34 (100.0%) | 51 (98.1%) | 85 (98.8%) |  |
| - Present | 0 (0.0%) | 1 (1.9%) | 1 (1.2%) |  |
| Pelvic pain |  |  |  | 0.851 ^1^ |
| - Absent | 31 (91.2%) | 48 (92.3%) | 79 (91.9%) |  |
| - Present | 3 (8.8%) | 4 (7.7%) | 7 (8.1%) |  |
| Trigeminal Neuralgia |  |  |  | 0.328 ^1^ |
| - Absent | 32 (94.1%) | 51 (98.1%) | 83 (96.5%) |  |
| - Present | 2 (5.9%) | 1 (1.9%) | 3 (3.5%) |  |
| Ankylosing spondylitis |  |  |  | 0.416 ^1^ |
| - Absent | 34 (100.0%) | 51 (98.1%) | 85 (98.8%) |  |
| - Present | 0 (0.0%) | 1 (1.9%) | 1 (1.2%) |  |
| Chronic fatigue |  |  |  | 0.335 ^1^ |
| - Absent | 31 (91.2%) | 50 (96.2%) | 81 (94.2%) |  |
| - Present | 3 (8.8%) | 2 (3.8%) | 5 (5.8%) |  |
| Polyarthralgia |  |  |  | 0.247 ^1^ |
| - Absent | 34 (100.0%) | 50 (96.2%) | 84 (97.7%) |  |
| - Present | 0 (0.0%) | 2 (3.8%) | 2 (2.3%) |  |
| Leg pain |  |  |  | 0.759 ^1^ |
| - Absent | 33 (97.1%) | 51 (98.1%) | 84 (97.7%) |  |
| - Present | 1 (2.9%) | 1 (1.9%) | 2 (2.3%) |  |
| Shoulder pain |  |  |  | 0.823 ^1^ |
| - Absent | 33 (97.1%) | 50 (96.2%) | 83 (96.5%) |  |
| - Present | 1 (2.9%) | 2 (3.8%) | 3 (3.5%) |  |
| Hip pain |  |  |  | 0.416 ^1^ |
| - Absent | 34 (100.0%) | 51 (98.1%) | 85 (98.8%) |  |
| - Present | 0 (0.0%) | 1 (1.9%) | 1 (1.2%) |  |
| Esophageal pain |  |  |  | 0.416 ^1^ |
| - Absent | 34 (100.0%) | 51 (98.1%) | 85 (98.8%) |  |
| - Present | 0 (0.0%) | 1 (1.9%) | 1 (1.2%) |  |
| Chest pain |  |  |  | 0.416 ^1^ |
| - Absent | 34 (100.0%) | 51 (98.1%) | 85 (98.8%) |  |
| - Present | 0 (0.0%) | 1 (1.9%) | 1 (1.2%) |  |
| Periumbilical |  |  |  | 0.214 ^1^ |
| - Absent | 33 (97.1%) | 52 (100.0%) | 85 (98.8%) |  |
| - Present | 1 (2.9%) | 0 (0.0%) | 1 (1.2%) |  |
| Rheumatoid arthritis |  |  |  | 0.416 ^1^ |
| - Absent | 34 (100.0%) | 51 (98.1%) | 85 (98.8%) |  |
| - Present | 0 (0.0%) | 1 (1.9%) | 1 (1.2%) |  |
| Complex regional pain syndrome |  |  |  | 0.416 ^1^ |
| - Absent | 34 (100.0%) | 51 (98.1%) | 85 (98.8%) |  |
| - Present | 0 (0.0%) | 1 (1.9%) | 1 (1.2%) |  |
| Condition sum |  |  |  | 0.761^2^ |
| - Mean (SD) | 2.059 (1.205) | 1.981 (1.129) | 2.012 (1.153) |  |
| - Range | 1.- 5 | 1 - 6 | 1. - 6 |  |

*Note:* ^1^ P-value obtained from Pearson’s Chi-squared test; ^2^LP-value obtained from linear Model ANOVA.
